# Pharmacological Primary Prevention of Diabetes Mellitus Type II: A Narrative Review

**DOI:** 10.7759/cureus.10033

**Published:** 2020-08-25

**Authors:** Ali Naveed, Larabe Farrukh, Muhammad Khawar Sana, Bazigh Naveed, Fawad Ahmad Randhawa

**Affiliations:** 1 Internal Medicine, King Edward Medical University & Mayo Hospital, Lahore, PAK; 2 Diabetes and Endocrinology, King Edward Medical University & Mayo Hospital, Lahore, PAK

**Keywords:** primary prevention, pharmacological prevention, diabetes mellitus type ii, pre-diabetes

## Abstract

The evolving epidemic of type 2 diabetes mellitus has challenged health-care professionals. It stands among the leading causes of mortality in the present world. It warrants new and versatile approaches to improve mortality and the associated huge quality-adjusted life years lost to it once diagnosed. A possible venue to lower the incidence is to assess the safety and efficacy of various diabetes prevention strategies. Diet and exercise have a well-developed role in the prevention of weight gain and, ultimately, diabetes mellitus type II in high-risk individuals. However, high-risk individuals can also benefit from adjunct pharmacotherapy. In light of this information, we decided to conduct a systematic review of randomized controlled trials. This article summarizes the evidence in the literature on the pharmacological prevention of diabetes in high-risk individuals.

## Introduction and background

Scientific progress, plentiful food, sedentary lifestyles, and financial evolution in the late 20th century introduced us to the obesity pandemic [1]. Obesity leads to increased fat deposition in the body, leading to increased insulin resistance and the widespread prevalence of diabetes mellitus type II [2]. It is projected that diabetes mellitus will increase by 38% by 2030[3], making it one of the biggest medical challenges of the 21st century. Diabetes mellitus type II is a chronic medical condition that leads to insulin resistance and the inability of uptake of glucose by the body storage cells[4]. This leads to the deposition of glucose in blood vessels, the lens of the eye, and nerves, leading to complications in almost every body system and causing a wide variety of macrovascular and microvascular complications [5] and overall increased mortality [6]. According to the Centers for Disease Control and Prevention (CDC) estimates, about 13.5% of the US adult population in 2018 had diagnosed diabetes mellitus type II and a further 2.6% adults had high blood sugar levels but were never diagnosed [7]. It is projected that by the end of 2030, a staggering 366-million of the human population would have been diagnosed with diabetes mellitus across the globe [8]. Diabetes mellitus type II clinically follows a very predictable progression, initially beginning with impaired glucose tolerance before full-blown diabetes [9]. The use of oral anti-glycemic agents and insulin has helped control blood glucose levels over the decades [10]. Despite worldwide availability and the widespread use of various medications to control diabetes mellitus, complications like neuropathy, amputations, cataracts, nephropathy, and retinopathy are on the rise [11]. This has led endocrinologists to work on the primary prevention of the disease and delay the onset of diabetes to improve the overall quality of life. Lifestyle modifications, including exercise and weight loss, have been studied in detail and proven to delay the onset and progression to diabetes mellitus type II [12]. However, one research estimates as much as 56% non-compliance to lifestyle modifications among diabetics[13]. Even though weight loss is effective in reducing the conversion of prediabetes to type 2 diabetes, it is difficult to achieve and maintain. Realistically, it may not be possible to apply these findings to larger cohorts and maintain these lifestyle changes for the long term. This has led us to the consideration of pharmacotherapy.

The pharmacological treatment of impaired glucose tolerance (IGT) with oral anti-glycemic agents has been shown to slow down the progression of IGT to type 2 diabetes more uniformly. The purpose of this review is to study the efficacy of various pharmacological modalities in the reduction of type 2 diabetes prevalence in high-risk populations.

## Review

Materials and methods

Objectives

The review was done to study the pharmacological approaches used in the prevention of diabetes mellitus type II over the years. The literature on the primary prevention of diabetes mellitus using different medications is limited, and an inclusive review with all such approaches will be a useful read for endocrinologists and internists alike to devise mechanisms to control the incidence of diabetes mellitus type II. *Databases*

The literature review for the research was performed on PubMed, Cochrane, and Clinicaltrials.gov [14]. MeSH terms type II diabetes mellitus, primary prevention, and pharmaceutical preparations were searched with all corresponding keywords, and relevant articles were imported into Endnote. Additionally, we searched databases using individual diabetic medications to make sure we do not miss any articles. All keywords are shown in Table 1.

**Table 1:** MeSH table for literature review

	Population/Problem ( Diabetes Mellitus type 2)	Intervention (Pharmacological Medications)	Outcome (Prevention)
Mesh term	Diabetes Mellitus, Type 2	Pharmaceutical Preparations	Primary prevention
Entry terms	Diabetes Mellitus, Ketosis Resistant; Ketosis-Resistant Diabetes Mellitus; Non-Insulin-Dependent Diabetes Mellitus; Diabetes Mellitus, Stable; NIDDM; Maturity-Onset Diabetes Mellitus; Maturity Onset Diabetes Mellitus; MODY; Diabetes Mellitus, Slow Onset; Slow-Onset Diabetes Mellitus; Type 2 Diabetes Mellitus Noninsulin-Dependent Diabetes Mellitus; Noninsulin Dependent Diabetes Mellitus; Diabetes, Maturity-Onset; Maturity Onset Diabetes; Diabetes, Type 2; Adult-Onset Diabetes Mellitus; Diabetes Mellitus, Adult Onset	Preparations, Pharmaceutical; Pharmaceutic Preparations; Preparations, Pharmaceutic; Pharmaceutical Products; Products, Pharmaceutical; Pharmaceuticals Drugs	Disease Prevention, Primary; Disease Preventions, Primary; Primary Disease Prevention; Primary Disease Preventions; Prevention, Primary; Primordial Prevention; Preventions, Primordial; Primordial Preventions; Prevention, Primordial

Inclusion Criteria

Described below are the inclusion criteria for our review: 1: All studies with nondiabetic and prediabetics participants 2: Observational and experimental studies 3: Good or fair-quality studies on the quality assessment questionnaire as shown in Table 2 4: Studies published in the English language

Exclusion Criteria

Described below are the exclusion criteria for our review: 1: Review studies 2: Poor-quality studies on the quality assessment questionnaire 3: Studies published in languages other than English 4: Studies that significantly include patients already diagnosed with type II diabetes mellitus 5: Editorials, letters to editors, and comments 6: Ongoing clinical trials *Study Selection*

**Figure 1:**
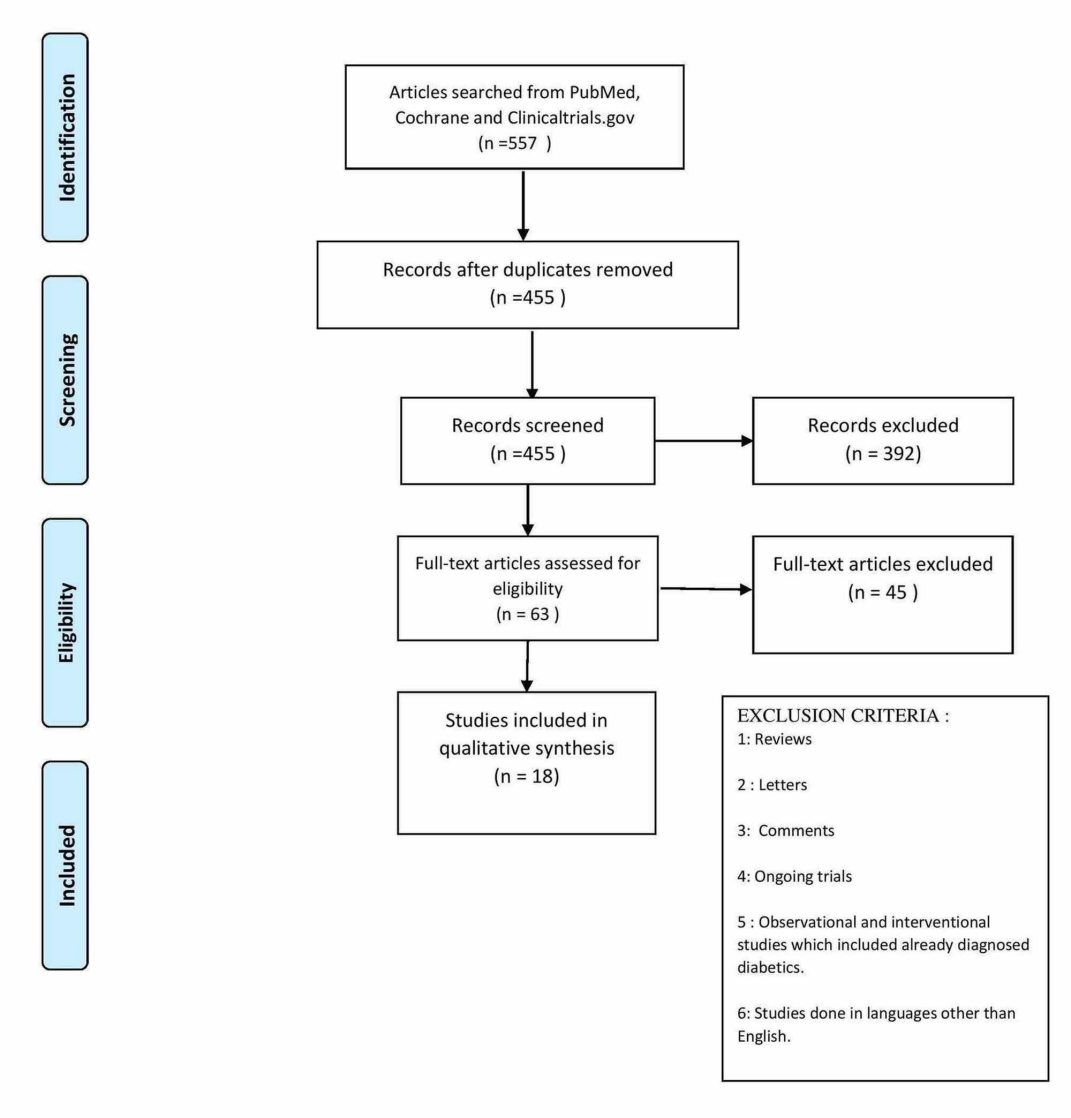
Screening and selection process for review

A total of 557 studies were imported into Endnote from the three databases. A total of 102 repeat articles were removed. Inclusion and exclusion criteria were applied and a total of 63 (13.8%) articles were selected after going through titles and abstracts. These 63 full articles were extracted and independently passed through the quality assessment questionnaire to finally select a total of 18 (30%) articles that have been included in the final review. Figure 1 shows the Preferred Reporting Items for Systematic Reviews and Meta-Analyses (PRISMA) flow chart for study selection [15].

**Table 2:** Quality assessment of included studies

Q. No	Ramachandran et al. [16]	Zinman et al. [17]	Sussmann et al. [18]	AlFawaz et al. [19]	Ariel et al. [20]	Le Roux et al. [21]	Guardado Mendoza et al. [22]	Chiasson et al. [23]	Nijpels et al. [24]	Koyasu et al. [25]	Kawamori et al. [26]	Hymsfield et al. [27]	Torgerson et al. [28]	Navigator study group [29]	Pittas et al. [30]	Durbin et al. [31]	Punthakee et al. [32]	Buchanan et al. [33]
1. Was the research question or objective in this paper clearly stated?	Yes	Yes	Yes	Yes	Yes	Yes	Yes	Yes	Yes	Yes	Yes	Yes	Yes	Yes	Yes	Yes	Yes	Yes
2. Was the study population clearly specified and defined?	Yes	Yes	Yes	Yes	Yes	Yes	Yes	Yes	Yes	Yes	Yes	Yes	Yes	Yes	Yes	Yes	Yes	Yes
3. Was the participation rate of eligible persons at least 50%?	Yes	Yes	Yes	Yes	Yes	Yes	Yes	Yes	Yes	Yes	Yes	Yes	Yes	Yes	Yes	Yes	Yes	Yes
4. Were all the subjects selected or recruited from the same or similar populations (including the same time period)? Were inclusion and exclusion criteria for being in the study prespecified and applied uniformly to all participants?	Yes	Yes	Yes	Yes	Yes	Yes	Yes	Yes	Yes	Yes	Yes	Yes	Yes	Yes	Yes	Yes	Yes	Yes
5. For the analyses in this paper, were the exposure(s) of interest measured prior to the outcome(s) being measured?	No	No	No	No	No	No	No	No	No	No	No	No	No	No	No	No	No	No
6. Was the timeframe sufficient so that one could reasonably expect to see an association between exposure and outcome if it existed?	Yes	Yes	Yes	Yes	Yes	Yes	Yes	Yes	Yes	Yes	Yes	Yes	Yes	Yes	Yes	Yes	Yes	Yes
7. For exposures that can vary in amount or level, did the study examine different levels of the exposure as related to the outcome (e.g., categories of exposure, or exposure measured as a continuous variable)?	No	No	No	No	No	No	No	No	No	No	No	No	No	No	No	No	No	No
8. Were the exposure measures (independent variables) clearly defined, valid, reliable, and implemented consistently across all study participants?	Yes	Yes	Yes	Yes	Yes	Yes	Yes	Yes	Yes	Yes	Yes	Yes	Yes	Yes	Yes	Yes	Yes	Yes
9. Was the exposure(s) assessed more than once over time?	No	No	No	No	No	No	No	No	No	No	No	No	No	No	No	No	No	No
10. Were the outcome measures (dependent variables) clearly defined, valid, reliable, and implemented consistently across all study participants?	Yes	Yes	Yes	Yes	Yes	Yes	Yes	Yes	Yes	Yes	Yes	Yes	Yes	Yes	Yes	Yes	Yes	Yes
11. Were the outcome assessors blinded to the exposure status of participants?	No	No	No	No	No	No	No	No	No	No	No	No	No	No	No	No	No	No
12. Was loss to follow-up after baseline 20% or less?	Yes	Yes	Yes	Yes	Yes	Yes	Yes	Yes	Yes	Yes	Yes	Yes	Yes	Yes	Yes	Yes	Yes	Yes
13. Were key potential confounding variables measured and adjusted statistically for their impact on the relationship between exposure(s) and outcome(s)?	Yes	Yes	Yes	Yes	Yes	Yes	Yes	Yes	Yes	Yes	Yes	Yes	Yes	Yes	Yes	Yes	Yes	Yes
Quality Rating	Good	Good	Good	Good	Good	Good	Good	Good	Good	Good	Good	Good	Good	Good	Good	Good	Good	Good

Data Extraction

Data extraction was done from selected studies in a tabulated form. The first author name, country of research, year of research, mean characteristics and size of the sample, the drug studied, and results were documented in the tabulated form shown in Table 3.

**Table 3:** Summarized table of the results derived from relevant studies HbA1c: glycated hemoglobin

Study	Country of research	Year of publication	Treatment drug used	Sample size (N) & gender distribution	Duration of study	Summary conclusions and results
Ramachandran et al. [16]	INDIA	2006	Metformin	531; 79% M, 21% F	3 years	Metformin and lifestyle modifications independently cause an equivocal decreased incidence of diabetes in high-risk patients. However, there is no additive effect of both therapies combined.
Zinman et al. [17]	CANADA	2006	Metformin and Rosiglitazone	200 (Gender % not mentioned)	4 years	The absolute risk reduction for the incidence of diabetes mellitus type II is 26%. The decreased incidence of the disease in the treatment group is statistically significant. Achievement of normal glucose tolerance is also statistically significant in the treatment group.
Sussmann et al. [18]	USA	2015	Metformin	3081; 33% M, 67% F	3 years	The sample was stratified into groups on the basis of risk factors for the development of diabetes mellitus. The Metformin effect was unevenly distributed in different groups. The treatment was most effective in the highest risk patients and not very effective in low-risk patients.
AlFawaz et al. [19]	KSA	2018	Metformin	294; 66% M, 34% F	1 year	Metformin and intense lifestyle modifications are almost equivocal in the decrease in the incidence of diabetes mellitus in high-risk patients. However, metformin causes a statistically significant greater weight loss than lifestyle modifications alone.
Ariel et al. [20]	USA	2014	Liraglutide	50; 55% M, 45% F	14 weeks	Liraglutide treatment led to a statistically significant decrease in weight loss and fasting blood glucose levels as compared to the placebo group without any severe side effects seen in the treatment group.
Le Roux et al. [21]	IRELAND	2017	Liraglutide	2254; 24% M, 76% F	3 years	Liraglutide caused a statistically significant decrease in the onset of type II diabetes mellitus. It also reverted prediabetics to normal glycemic levels. After remission of therapy, 50% of the reverted patients got back to the prediabetic state while the remaining 50% continued to be normoglycemic and the conclusion is statistically significant.
Guardado-Mendoza et al. [22]	MEXICO	2019	Linagliptin plus Metformin vs Metformin alone	144 (Gender % not mentioned)	2 years	Linagliptin plus Metformin combination produced greater effects as compared to Metformin alone and caused a statistically significant decrease in fasting blood glucose(p<0.05) and HbA1c9p<0.01). Side effects were similar in both groups and very mild.
Chiasson et al. [23]	CANADA	2002	Acarbose	1429; 49% M, 51% F	3 years	The study concluded that participants on acarbose were 25% less likely to proceed to diabetes vs. placebo. 32.4% of participants in the acarbose arm developed diabetes at two years follow-up compared to 41.5% in the placebo arm. Gastrointestinal side effects were noted in the treatment arm.
Nijpels et al. [24]	NETHERLANDS	2008	Acarbose	118; 50% M, 50% F	3 years	There was a statistically significant decrease in the blood glucose levels and incidence of diabetes in the treatment arm. However, there was a 30% dropout in the treatment arm due to abdominal pain and other gastrointestinal side effects.
Koyasu et al. [25]	JAPAN	2010	Acarbose	90; 91% M, 9% F	1 year	Intimal media thickening was reduced in patients in the treatment arm with p<0.001 and reduction in OGTT test values was also statistically significant. No severe adverse effects were reported.
Kawamori et al. [26]	JAPAN	2009	Voglibose	1780; 60% M, 40% F	3 years	The incidence of type II diabetes mellitus was significantly reduced in the treatment arm with p=0.0014. Significant prediabetics reverted back to normal glucose levels. Side effects like gastrointestinal disturbances including flatulence, abdominal distention, and diarrhea, were observed in 90% of patients in the treatment arm vs 85% of patients in the placebo arm. 5% of the patients in the treatment arm discontinued therapy.
Hymsfield et al. [27]	USA	2000	Orlistat	675; 20% M, 80% F	2 years	Orlistat caused a statistically significant weight loss in the treatment arm leading to only 3% of patients developing diabetes during or at the end of the study as compared to more than 7% in the placebo arm.
Torgerson et al. [28]	USA	2004	Orlistat	3305; 55% M, 45% F	4 years	Orlistat led to statistically significant (p=0.0032) risk reduction in the occurrence of diabetes mellitus in the treatment group as compared to placebo as well as a reduction in weight loss (p<0.001). 90% of people in the treatment group experienced mild GI symptoms compared to 65% in the placebo group but only 8% dropped out of treatment in the orlistat group.
Holman et al. [29]	ENGLAND	2010	Nateglinide	9306; 49% M, 51% F	5 years	The large trial concluded that nateglinide was unable to reduce the incidence of diabetes mellitus type II in the high-risk populations. However, nateglinide was associated with a mildly increased risk of hypoglycemia.
Pittas et al. [30]	USA	2019	Vitamin D	2423; 60% M, 40% F	2 years	The large randomized trial showed that there Is no statistically significant reduction in the incidence of diabetes mellitus in high-risk populations when they receive vitamin D. The study showed a mild increase in hypercalcemia, nephrolithiasis, and associated side effects of hypercalcemia but the findings were statistically insignificant.
R.J. Durbin [31]	USA	2004	Rosiglitazone	172; 50% M, 50% F	3 years	The study concluded that thiazolidinediones were effective in reducing the incidence of diabetes in high-risk populations. It was estimated that treatment of 4.2 high-risk people with a glitazone for 3 years will prevent the occurrence of diabetes in one patient (p<0.001)
Punthakee et al. [32]	CANADA	2014	Rosiglitazone	190; 45% M, 55% F	3.5 years	Rosiglitazone lowered fasting glucose by 0.36 mmol/l more vs. placebo [95% CI 0.16–0.56] (p=0.0004). Similarly, two-hour postprandial glucose decreased by 1.21 mmol/l [95% CI 0.51–1.91] vs. placebo (p=0.0008). The study further reported that this effect on glucose was independent of the effect of rosiglitazone on adiponectin, total body, visceral, hepatic, or subcutaneous fat.
Buchanan et al. [33]	USA	2002	Troglitazone	266; 100% F	2.5 years	The study concluded that troglitazone reduces the incidence of diabetes high-risk women by 50%. Statistically significant reduction in the incidence of diabetes mellitus was seen in Hispanic women. Hepatotoxicity was seen in 8 patients who received troglitazone. The drug was later withdrawn from the market due to poor side-effect profile.

Results

Metformin

Metformin is the drug most studied for the primary prevention of diabetes mellitus in high-risk patients. In our review, we considered four randomized control trials that studied the effect of metformin. The studies show an average absolute risk reduction of 16% among the participants who received metformin across all those trials. The Indian Diabetes Prevention Programme was a community-based trial that studied the influence of intervention in the incidence of diabetes [16]. The subjects were individuals with impaired glucose tolerance (IGT) who were treated with lifestyle modifications (LSM), metformin (MET), and a combination of both LSM and metformin. Lifestyle modifications and metformin showed a similar reduction in the incidence of diabetes mellitus type II over the 30 months of trial, however, the combination of lifestyle modifications and metformin did not show any added benefits as compared to lifestyle modifications and metformin alone. None of the side effects were statistically significant except for the gastrointestinal side effects that occurred in 30 patients taking metformin. On reporting of side effects, the metformin dose was reduced in those patients after which no side effects were reported.

The Canadian Normoglycemia Outcomes Evaluation (CANOE) trial investigated the effect of combination pharmacological therapy on the development of diabetes mellitus type II [17]. Participants included patients with IGT who were allocated into two groups. The first group received combination rosiglitazone (2 mg) and metformin (500 mg) twice daily while the second group received matching placebo for a median of 3·9 years. The incidence of diabetes was significantly reduced in the active treatment group (n=14 (14%)) as compared to the placebo group (n=41 (39%); p<0·0001). The relative risk reduction was 66% (95% CI 41-80) and the absolute risk reduction was 26% (14-37). Around 80% of patients in the treatment group regressed to normal glucose tolerance as compared with only 53% in the placebo group (p=0·0002). Furthermore, by study end, insulin sensitivity had decreased to a greater extent in the placebo group than in the rosiglitazone and metformin group. The study did not report any side effects experienced by the patients.

Sussmann et al. studied the occurrence of diabetes in subjects who were stratified by their risk of developing diabetes according to a diabetes risk prediction model [18]. In this study, 3081 participants with impaired glucose metabolism at baseline were taken. It was observed that the benefit of metformin was distributed quite unevenly across the study population. Participants who were at the highest likelihood of having diabetes in the future received far greater benefits from metformin therapy (21.5% absolute reduction in diabetes over three years of treatment) while patients who were in the lower risk group achieved little or no benefit. In the lowest risk quarter for progression to diabetes, the Metformin group had a slightly higher risk of developing diabetes (9.6%) than did the control group (8.3%). In the highest risk quarter, the metformin group had an absolute risk reduction of 21.4% while the control group had a 59.6% observed rate of developing diabetes. Therefore, patients with the greatest risk of developing diabetes had a statistically significant relative risk reduction from metformin use (p<0.001). Therefore, this type of benefit-based tailored treatment, using a multivariable risk prediction tool, could decrease drug overuse and help make the prevention of diabetes far more efficient, effective, and patient-centered. The study did not report the side effects experienced by the patients.

Alfawaz et al. studied the status of metabolic syndrome in individuals receiving the Intensive Lifestyle Modification Programme (ILMP) or low dose metformin as compared to the control group [19]. The occurrence of metabolic syndrome in the ILMP group decreased by 26% (p<0.001), in the metformin group by 22.4% (p=0.013), and the control group by 8.2% (p=0.281). Mean fasting glucose was significantly reduced in the ILMP and metformin groups while in the control group, this reduction was modest. Similarly, hyperglycemia also decreased significantly by 38.4% in the ILMP group and 39% in the metformin group, respectively. The mean reduction in fasting glucose in the ILMP group is less than that found in the metformin group. In the study, the mean weight loss from baseline to the end of the study was 4.15 kg in the metformin group as compared to 1.6 kg in the ILMP group. The study did not document the side effects that were experienced by the patients who had metformin.

*Liraglutide*

Ariel et al. were the first to study the effect of liraglutide on a small number of prediabetic patients [20]. Twenty-three patients out of 50 received the medication while 27 received a placebo. Weight loss was greater in the liraglutide group as compared to the placebo group (6.9 vs. 3.3 kg, p<0.001) and so was the decrease in fasting blood glucose level (9.9 mg/dL vs. 0.3 mg/dL, p<0.001). This study showed the potential for the use of liraglutide in the prevention of diabetes mellitus type II. A three-year study was designed to observe the effect of liraglutide in combination with diet and exercise to decrease the risk of developing diabetes mellitus Type II in prediabetic individuals [21]. Liraglutide was associated with a risk reduction in the onset of type 2 diabetes by 80% as compared to placebo (HR 0·21, 95% CI 0·13-0·34). By week 160, 2% of individuals in the liraglutide group versus 6% in the placebo group were diagnosed with diabetes while on treatment. The time to onset of diabetes over 160 weeks among all groups was 2·7 times longer with liraglutide than with placebo (95% CI 1·9 to 3·9, p<0·0001). After 160 weeks, regression from prediabetes to normoglycemia was observed in 66% of individuals in the liraglutide group (odds ratio (OR) 3·6, 95% CI 3·0-4·4, p<0·0001). Liraglutide-induced greater weight loss was more significant compared to the placebo at week 160 (-6·1% for liraglutide vs -1·9% for placebo; estimated treatment difference -4·3%, 95% CI -4·9 to -3·7 (p<0.0001). Weight loss with liraglutide treatment was sustained over three years. Insulin resistance and β-cell function improved in the liraglutide group as compared with the placebo group at week 160 and glycated hemoglobin, fasting glucose, and fasting insulin concentrations were also lower with liraglutide. After 12-week treatment cessation, some individuals in the liraglutide group reverted to prediabetes but 50% of the treated individuals still had normoglycemia at week 172 as compared with 36% of the individuals in the placebo group (p<0·0001).

Liraglutide has a well-documented safety profile and is well-tolerated. Some gastrointestinal disorders, notably nausea, diarrhea, constipation, and vomiting, were observed in the liraglutide group as compared to the placebo group.

Linagliptin

The PRELLIM (Diabetes Prevention with Linagliptin, Lifestyle, and Metformin) project was designed to study the efficacy of a combination of linagliptin + metformin + lifestyle modification as compared to metformin + lifestyle modification only [22]. At 24 months, glucose levels remained improved in both groups but significantly more in the LM group mainly at 60 minutes in the oral glucose tolerance test (OGTT) (167±3 vs 155±3, in M and LM group, respectively, p<0.05). Insulin sensitivity measured from OGTT and during fasting had more pronounced improvement in the LM group as compared to the M group (p<0.05). Reduction in glycated hemoglobin (HbA1c) was observed only in the LM group (p<0.01). Improvements in β-cell function at 24 months persisted in both groups but they were significantly better in the LM group (p<0.05). The probability to regress to normoglycemia and normal glucose tolerance was significantly higher in the LM group than in the M group during the entire follow-up (OR adjusted per month: 3.31, 95% CI: 1.5 4 -7.09, p=0.00 2). Side effects: Diarrhea occurred in six patients from the M group and in five patients from the LM group. Nausea occurred in two patients from each group, cephalea in two from the M group, and one from the LM group. *Acarbose*

Chiasson et al. (2002) (n=1429) studied the role of acarbose in delaying or preventing the progression of impaired glucose tolerance to full-blown type 2 diabetes mellitus [23]. In this multicenter placebo-controlled randomized trial, participants were randomized 1:1 to either arm and were followed up for 3.3 years. Participants on acarbose were 25% less likely to proceed to diabetes vs. placebo. In the acarbose arm, 32.4% of participants developed diabetes at the two-year follow-up as compared to 41.5% in the placebo arm. The study concluded that acarbose not only decreases progression from glucose impairment to diabetes but also brings the impairment back to normal. Ninety-eight percent (98%) of the acarbose group and 95% of the placebo group suffered from at least one adverse event. The most common adverse events were gastrointestinal and were significantly higher in the acarbose arm than the placebo (p<0.0001), but they were considered mild to moderate in intensity. The common adverse events were diarrhea, flatulence, and abdominal pain, all of which were higher in the acarbose group.

Nijpels et al. (2008) (n=118) studied the metabolic effects of acarbose on glucose in individuals who are at high risk of developing diabetes [24]. The dropout rate was high, with 52 participants leaving the trial, 30 of whom left due to adverse events. The mean difference in plasma glucose levels after three years of treatment was −1.16 mmol/L (95% CI: −2.03; −0.17). Twenty-four point one percent (24.1%) participants converted to diabetes in the placebo arm vs. 18.3% in the acarbose arm (Relative Risk: 0.76 (95% CI: 0.38; 1.53)). The study concluded that the absolute risk reduction with acarbose for developing diabetes was 6% (95% CI: −9; 21). Almost one-third (36.7%) of the participants in the acarbose arm left the trial due to adverse events vs. only 13.6% in the placebo arm. Acarbose had significantly higher adverse events, the majority of which were related to the gastrointestinal system. Thirteen point one (13.1%) of participants reported abdominal pain in the acarbose arm vs. 3.3% in the placebo. Similarly, diarrhea was reported by 19.7% in the acarbose arm vs. 1.7% in the placebo. Flatulence had the most significant difference in incidence with 44.3% in acarbose vs. 3.3% in placebo. Koyasu et al. (2010) (n=90) randomized the participants with impaired glucose tolerance or early diabetes in a 1:1 ratio to either receive acarbose or placebo [25]. The primary endpoint was the absolute change in intima-media thickness (IMT) of the carotid artery at the one-year follow-up. The mean change in IMT was 0.02 mm in the acarbose group vs. 0.17 mm in the placebo (p=0.01). Furthermore, among several secondary endpoints, only two-hour 75 g OGTT and at one year in the acarbose arm had significant reductions in OGGT results from 192.8 to 168.6 mg/dL; mean change -24.8 mg/dL (p=0.001). There was no significant reduction in HbA1c in either group. However, the control arm had a significant rise in fasting immunoreactive insulin from 7.17 to 8.58 μU/mL; mean change +1.30 μU/mL (p=0.02). There were four patients in each group with cardiovascular events requiring hospitalizations. No other adverse events were reported.

Voglibose

Kawamori et al. (2009) (n=1778) studied the role of voglibose in the prevention of type 2 diabetes mellitus [26]. In this double-blinded, randomized, placebo-controlled trial, participants were to be followed for three years. The interim analysis showed significant improvement in the voglibose group, hence leading to early termination of the trial. At the end of the study, a significantly fewer number of participants had developed full-blown diabetes in the voglibose group vs. the placebo group (50 of 897 vs. 106 of 881; hazard ratio 0.595 (95% CI 0.433-0.818); p=0.0014). Similarly, more people reverted to normal glucose levels in the voglibose group than in the placebo group (599 of 897 vs. 454 of 881; hazard ratio 1.539 (95% CI 1·357-1·746); p<0.0001). Ninety percent (90%) of the experimental arm were reported to have suffered adverse events vs. only 85% in the control group (p=0.0009). Five percent of participants from the voglibose arm vs. 3% of participants from the control arm discontinued the therapy due to adverse events in which causality could not be denied (p=0.0092). Though only mild to moderate in severity, most common adverse events in voglibose vs. placebo were flatulence 17% vs. 7%, abdominal distention 13% vs. 5%, and diarrhea 13% vs. 5%, respectively. A total of six deaths were reported in the voglibose arm, none of which were related to drug treatment (two were attributed to accidents and one each to heart failure due to self-intoxication, myocardial infarction, lung cancer, suicide). No deaths were reported in the placebo group.

Orlistat

Heymsfied et al. (2000) (n=675) did a retrospective meta-analysis to study the effect of weight loss from orlistat on glucose tolerance and progression to type 2 diabetes in obese adults [27]. Results from three randomized, double-blinded, placebo-controlled, multicenter trials were pooled. The mean duration was 582 days. Participants in the orlistat group lost more weight than in the placebo, mean ± SEM; 6.81 ± 0.41 kg vs. 3.79 ± 0.38 kg, respectively (p<0.001). In participants with impaired glucose tolerance, the orlistat group achieved normoglycemia in 71.6% vs. 49.1% in the placebo group (p=0.04). Moreover, only 3% of participants from the orlistat arm converted to diabetes vs. 7.6% from the placebo arm. No adverse event data were reported. The study concluded that orlistat, through weight loss, significantly lowers the risk of deterioration of glucose tolerance and even reverts to normal levels in high-risk obese adults.

Torgerson et al. (2004) (n=3305) conducted XENDOS (XENical in the Prevention of Diabetes in Obese Subjects), a randomized study of orlistat effects as an adjunct to lifestyle modification in the prevention of type 2 diabetes in obese adults [28]. In the intention-to-treat analysis, 52% of the orlistat group completed the treatment vs. 34% of the placebo (p=<0.0001). At the four-year follow-up, 9% of the placebo group participants vs. 6.2% of the orlistat group participants developed diabetes (risk reduction: 37.3%; p=0.0032). Subanalysis revealed that this difference was due to subjects with impaired glucose tolerance in the respective group. Mean weight loss with orlistat was 5.8 kg vs. 3 kg with placebo (p<0.001). The inclusion of dropouts into the analysis still revealed significant weight loss in the orlistat group 3.6 kg vs. 1.4 kg (p<0.001). Orlistat was associated with a significantly higher incidence of gastrointestinal adverse events vs. placebo. The incidence of all other adverse events was similar in both groups. Most of these adverse events were mild to moderate. Ninety-one percent (91%) of the orlistat group reported adverse events vs. 65% of the placebo group in the first year of treatment. With time, the incidence reduced to 35% vs. 23% in orlistat vs. placebo at the four-year follow-up. Overall, 8% vs. 4% of participants stopped treatment due to adverse events in the orlistat vs. placebo groups, respectively. No deaths were attributed to orlistat. The study concluded that orlistat significantly improves the outcome in obese adults who are at risk of developing diabetes when used in adjunct with lifestyle modifications.

Nateglinide

A study group called NAVIGATOR (Nateglinide and Valsartan in Impaired Glucose Tolerance Outcomes Research) conducted a large, double-blinded, randomized clinical trial (2010) (n=9306) where the participants with impaired glucose tolerance were randomized into 2 x 2 factorial design to either receive nateglinide vs. placebo or valsartan vs. placebo, both in adjunct with lifestyle modifications [29]. About 69.9% and 71% of the nateglinide and placebo groups, respectively, completed treatment for the whole five years. Thirteen point one percent (13.1%) from the nateglinide group vs. 12.9% from the placebo group were lost to follow-up or withdrew while 11.2% vs. 10.4% from the respective group stopped treatment due to adverse events. At the five-year follow-up, the study reported that nateglinide did not reduce the incidence of diabetes significantly (36% vs. 34%, respectively; hazard ratio, 1.07; (95% CI 1.00 to 1.15); p=0.05. However, although mild, nateglinide did raise the hypoglycemia risk; 19.6% in nateglinide vs. 11.3% in the placebo group (p<0.001).

Vitamin D

Pittas et al. (2019) (n=2423) conducted a randomized clinical trial to validate the results of observational studies on whether vitamin D is associated with decreasing the risk of type 2 diabetes or not [30]. A 1:1 randomization was done for participants who fulfilled the criteria of prediabetes to either receive 4000 IU per day of vitamin D or placebo irrespective of their baseline serum concentration. The mean serum levels of 25-OH vitamin D at baseline were 27.7 ng/ml in the vitamin D group vs. 28.2 ng/ml in the placebo group. After two years, the levels rose to 54.3 ng/ml and 28.8 ng/ml. The 2.5-year follow-up showed that the 293/1211 participants in the vitamin D group developed diabetes vs. 323/1212 in the placebo group with a hazard ratio of 0.88 (95% CI 0.75 to 1.04), (p=0.12). A sensitivity sub-analysis after accounting for missing data did not change the results significantly. The study concluded that no significant reduction in risk to develop diabetes was noticed among prediabetics with the consumption of vitamin D when compared to placebo. Three-point nine percent (3.9%) of participants in the vitamin D group stopped treatment due to adverse events vs. 3.1% in the placebo (95% CI −0.7 to 2.3). Adverse events reported in the vitamin D vs. placebo groups were hypercalcemia (5 vs. 3), calcium:creatinine ratio>0.375 (1 vs. 1), low estimated glomerular filtration rate (eGFR) (1 vs. 2), patient-reported nephrolithiasis (25 vs. 21), serious adverse events (235 vs.228), and deaths (5 vs. 5). The incidence rate ratio of each of these adverse events in the vitamin D group vs. the placebo group was statistically insignificant.

Rosiglitazone

Durbin et al. (2004) (n=172) did a prospective analysis among individuals with impaired glucose tolerance who were initially taking troglitazone but later switched to rosiglitazone or pioglitazone when the troglitazone was withdrawn from the United States market due to toxicity reports [31]. One-hundred one (101) such individuals were identified. A control group of 71 individuals who had glucose impairment but were not taking any antidiabetic medication was included. At the three-year follow-up, 2.97% of participants in the rosiglitazone group progressed to diabetes while 26.6% of participants in the control group progressed to diabetes. The mean baseline HbA1c for rosiglitazone (6.12% ± 0.60) went down to 5.57% ± 0.37 after three years. Similarly, 6.23% ± 0.74 of pioglitazone went down to 5.65% ± 0.48. Paradoxically, the HbA1c in the placebo group went from a baseline of 6.18% ± 0.20 to 6.68% ± 0.19, an increase of 0.52% ± 0.16 from the baseline (p<0.001). The risk reduction after three years of follow-up was reported as 88.9% in the rosiglitazone group vs. the placebo (p<0.001). According to the study, treating 4.2 patients with either of the thiazolidinedione drugs would prevent one case of diabetes in a three-year duration. No adverse events data were collected during the study and all the patients completed the study.

Punthakee et al. (2014) (n=190) did a sub-analysis of the DREAM (Diabetes Reduction Assessment With Ramipril and Rosiglitazone Medication) trial where the role of rosiglitazone was studied on glucose, ectopic fat, adipokines, adiponectin, and fatty acids in population with impaired fasting glucose or glucose tolerance [32]. Eighty-eight (88) participants received rosiglitazone vs. 102 who received a placebo. Rosiglitazone lowered fasting glucose by 0.36 mmol/l more vs placebo (95% CI 0.16-0.56) (p=0.0004). Similarly, two-hour postprandial glucose decreased by 1.21 mmol/l (95% CI 0.51-1.91) vs. placebo (p=0.0008). The study further reported that this effect on glucose was independent of the effect of rosiglitazone on adiponectin, total body, visceral, hepatic, or subcutaneous fat.

Troglitazone was discontinued in the US market in the year 2000 after significant hepatotoxicity-related deaths were reported. But before the withdrawal, a trial was conducted between 1995 and 1998 by Buchanan et al. (2002) (n=266) to evaluate the prevention of type 2 diabetes using troglitazone in high-risk Hispanic women with previous gestational diabetes history and impaired glucose tolerance at enrollment [33]. A total of 30 participants were lost to follow-up (19 in the intervention group vs. 11 in the control group) all of whom did not differ significantly at baseline. During blind treatment, the annual incidence of diabetes remained at 12.1% vs. 5.4% in the placebo vs. troglitazone group, respectively (hazard ratio 0.45 (95% CI 0.25-0.83) and remained unchanged after adjustment for baseline characteristics. The study concluded that troglitazone reduces the incidence of diabetes in high-risk women by 50% at the minimum and offers long-term protection from it even after stopping the drug. Eight patients discontinued the medication due to raised serum hepatic enzymes. They resumed the drug after the enzymes returned to the baseline. One patient left the trial citing personal reasons.

Discussion

Diabetes mellitus type II is one of the leading factors of cardiovascular morbidity and mortality, which is the most prevalent cause of death in the older population [34]. Diabetes mellitus also leads to multiple organ damage and is one of the leading causes of disability in vision, kidney function, and limbs in old age [35]. Diabetes mellitus and its complications have been a challenge for physicians all over the globe in the last century. After thousands of studies, it has been concluded that the best way to save mankind from the morbidity of diabetes is to prevent its occurrence [36]. Intense lifestyle modifications in high-risk individuals have always been presented as the best means to prevent diabetes mellitus type II. Numerous studies have talked about the benefits of exercise and diet restriction on not only diabetes mellitus but also overall better health of the individuals [37]. Watanabe et al. concluded that lifestyle modifications, diet, and exercise improve all the factors of metabolic syndrome [38]. However, from a practical point of view, things are not as simple as they look. Rise et al. conclude that the knowledge and awareness of the public about lifestyle modification have almost no effect at all on their change in diet and physical activity [39]. Numerous nutritionists and physicians have tried and failed to find ways and means to convince the public into following ways and means to change their lifestyle [40]. On the other hand, there is a steep rise in the use of off-the-label medications [41] both by physicians and online in the management of obesity, which brings with it a long list of side effects and complications. In this current status quo, much neglected are the different studies done in the past to prevent diabetes mellitus in a high-risk population. Most of these studies have compelling evidence that the use of certain medications in the primary prevention of diabetes mellitus is not only effective but also associated with only mild side effects. Sussmann et al. showed effective primary prevention of diabetes mellitus in a big sample of high-risk individuals by the use of metformin [18]. Le Roux et al. studied a large sample of the high-risk population and proved with evidence that liraglutide is effective in the primary prevention of diabetes mellitus type II in the high-risk population with minimal side effects [21]. Multiple studies have shown the effectiveness of the thiazolidinedione group of anti-glycemic medications in the primary prevention of diabetes mellitus type II with a mild or minimal side-effect profile [17,31-32]. In current settings, with the surge of diabetes diagnosis and more expected in the upcoming years and impracticality of lifestyle modifications in the current day and age, it is imperative that medical scientists look into the promising initial trials in the prevention of diabetes in at least high-risk populations and consider doing large-scale studies that lead to the formation of guidelines to prevent diabetes mellitus.

## Conclusions

Our review of the literature has shown that even modest changes in lifestyle can decrease the progression of impaired glucose tolerance to diabetes by 50%-60%. However, the incidence of progression is still very high. The adjunct use of pharmacotherapy can further reduce the progression to full-blown diabetes. Anti-glycemic drugs are effective in reducing the incidence of type 2 diabetes in high-risk populations. Large randomized controlled trials should be designed to further corroborate these findings.
